# Heat resilience in embryonic zebrafish revealed using an *in vivo* stress granule reporter

**DOI:** 10.1242/jcs.234807

**Published:** 2019-10-15

**Authors:** Ruiqi Wang, Hefei Zhang, Jiulin Du, Jin Xu

**Affiliations:** 1 Institute of Neuroscience, State Key Laboratory of Neuroscience, Shanghai Institutes for Biological Sciences, Chinese Academy of Sciences, Shanghai 200031, China; 2 CAS Center for Excellence in Brain Science and Intelligence Technology, Shanghai 200031, China

**Keywords:** Stress granule, G3BP1, *In vivo* reporter, Zebrafish, Heat shock, Stress resilience, Early development

## Abstract

Although the regulation of stress granules has become an intensely studied topic, current investigations of stress granule assembly, disassembly and dynamics are mainly performed in cultured cells. Here, we report the establishment of a stress granule reporter to facilitate the real-time study of stress granules *in vivo*. Using CRISPR/Cas9, we fused a green fluorescence protein (GFP) to endogenous G3BP1 in zebrafish. The GFP–G3BP1 reporter faithfully and robustly responded to heat stress in zebrafish embryos and larvae. The induction of stress granules varied by brain regions under the same stress condition, with the midbrain cells showing the highest efficiency and dynamics. Furthermore, pre-conditioning using lower heat stress significantly limited stress granule formation during subsequent higher heat stress. More interestingly, stress granule formation was much more robust in zebrafish embryos than in larvae and coincided with significantly elevated levels of phosphorylated eIF2α and enhanced heat resilience. Therefore, these findings have generated new insights into stress response in zebrafish during early development and demonstrated that the GFP–G3BP1 knock-in zebrafish could be a valuable tool for the investigation of stress granule biology.

This article has an associated First Person interview with the first author of the paper.

## Abstract (precis)

**Summary:** Establishment of a new transgenic zebrafish line with knock-in GFP-G3BP1 to visualize stress granule dynamics in live animals in real time.

## INTRODUCTION

Stress granules are cytoplasmic structures rich in mRNA and RNA-binding proteins. They are usually formed when translation initiation is inhibited. This inhibition could be caused by certain drugs, altered expression or modification of translation initiation factors, or dissociation of ribosomal mRNA (Buchan and Parker, 2009; Dang et al., 2006; Gilks et al., 2004; Kedersha et al., 2000; Mokas et al., 2009). Furthermore, as the name suggests, stress granules are induced upon various stress insults, such as heat shock, viral infection and increased oxidative or endoplasmic reticulum (ER) stress (Kedersha et al., 1999; Nover et al., 1989; Protter and Parker, 2016; White and Lloyd, 2012; Wolozin, 2012). The formation of stress granules is considered to be a protective cellular mechanism for resource conservation and survival under unfavorable conditions, and is characterized by the translation inhibition of most house-keeping genes and the preferential translation of pro-survival stress-responsive genes (Anderson and Kedersha, 2002; Kedersha et al., 2013; McCormick and Khaperskyy, 2017).

Stress granule formation is a dynamic process, with its assembly and disassembly regulated by the abundance of many RNA-binding proteins (Protter and Parker, 2016). Mounting evidence indicates that stress granule dysregulation could contribute to the development of some neurodegenerative diseases (Apicco et al., 2018; Ash et al., 2014; Li et al., 2013; Maziuk et al., 2017; Xu et al., 2019) and chemoresistance in cancer cells (Anderson et al., 2015). Recently, we have shown that stress granule formation is also regulated by circadian rhythm (Wang et al., 2019). Therefore, stress granules play important roles in human health and diseases and warrant in-depth investigation.

Most studies of stress granules have been performed in cultured cells by immunolabeling stress granule marker proteins in fixed cells, or by live imaging of fluorescent protein-tagged stress granule markers (Kedersha and Anderson, 2007; Kedersha et al., 2000, 2005, 2008). *In vivo* studies of stress granules have been attempted using immunofluorescence labeling of fixed tissues (Bai et al., 2016; Shelkovnikova et al., 2017; Wang et al., 2019). However, the spatial and temporal regulation of the stress granules and their dynamics under physiological or disease states are entirely unknown. A previous study using fluorescence-tagged RNA as a reporter has generated some clues on the RNA dynamics in *Drosophila* muscle cells (van der Laan et al., 2012). Nevertheless, the current knowledge about the dynamics of protein components in stress granules *in vivo* is absent.

Ras GTPase-activating protein-binding protein 1 (G3BP1) is one of the RNA-binding proteins that can initiate and promote stress granule formation (Tourrière et al., 2003). By binding untranslated mRNA and serving as a scaffolding protein, G3BP1 facilitates the recruitment of other stress granule components via aggregation-prone low complexity domains (Buchan, 2014; Mahboubi and Stochaj, 2017). G3BP1 has been commonly used as a stress granule marker protein (Mahboubi and Stochaj, 2017; Protter and Parker, 2016) and green fluorescent protein (GFP)-tagged G3BP1 is routinely used to study stress granule dynamics in live cells. However, as overexpression of G3BP1 could induce stress granules (Anderson and Kedersha, 2008; Mahboubi and Stochaj, 2017), monitoring stress granule formation with an overexpressed protein is not an ideal approach. Previously, we have established a knock-in cell line expressing GFP–G3BP1 under the endogenous G3BP1 promoter (Wang et al., 2019). In the current study, we successfully tagged endogenous zebrafish G3BP1 with GFP using CRISPR-Cas9 gene editing and validated GFP–G3BP1 to be a functional *in vivo* stress granule reporter. Furthermore, with this new tool, we have found that the efficiency and dynamics of stress granule formation differed in various brain regions, and that heat stress pre-conditioning blunted stress granule formation *in vivo*. Surprisingly, we have also found higher stress resilience in zebrafish embryos than in larvae during early development. Therefore, we have demonstrated that this novel knock-in GFP–G3BP1 reporter could be a highly useful tool to investigate stress granule regulation *in vivo*.

## RESULTS

### Establishment and characterization of an *in vivo* stress granule reporter

We reasoned that the ideal *in vivo* reporter of stress granule formation should have the following properties. First, the marker protein should be functionally conserved in various species. Second, the expression of reporter protein would not interfere with the physiological formation of stress granules. Third, the stress granules could be easily monitored in real-time, and the dynamics could be analyzed. The zebrafish (*Danio* *rerio*) has become a valuable tool in biological research to visualize physiological changes using live imaging, owing to its transparent body at embryonic and early larval stages (Cooper et al., 1999a,b; Kimmel, 1989; Kimmel and Warga, 1988; Solnica-Krezel et al., 1995; Spitsbergen and Kent, 2003). Thus, we decided to genetically knock in a GFP tag to the zebrafish G3BP1 protein, which shares 65% protein sequence homology with its human counterpart. With this approach, the expression of GFP-tagged G3BP1 was under the control of the endogenous *g3bp1* gene promoter, meaning that stress granule formation would not be affected by G3BP1 overexpression. Although stress granule biology has not been well characterized in zebrafish, several studies have shown the formation of cytosolic granules resembling stress granules either under stress or with the expression of neurotoxic, stress granule-inducing proteins (Bosco et al., 2010; Zampedri et al., 2016).

**Fig. 1.**
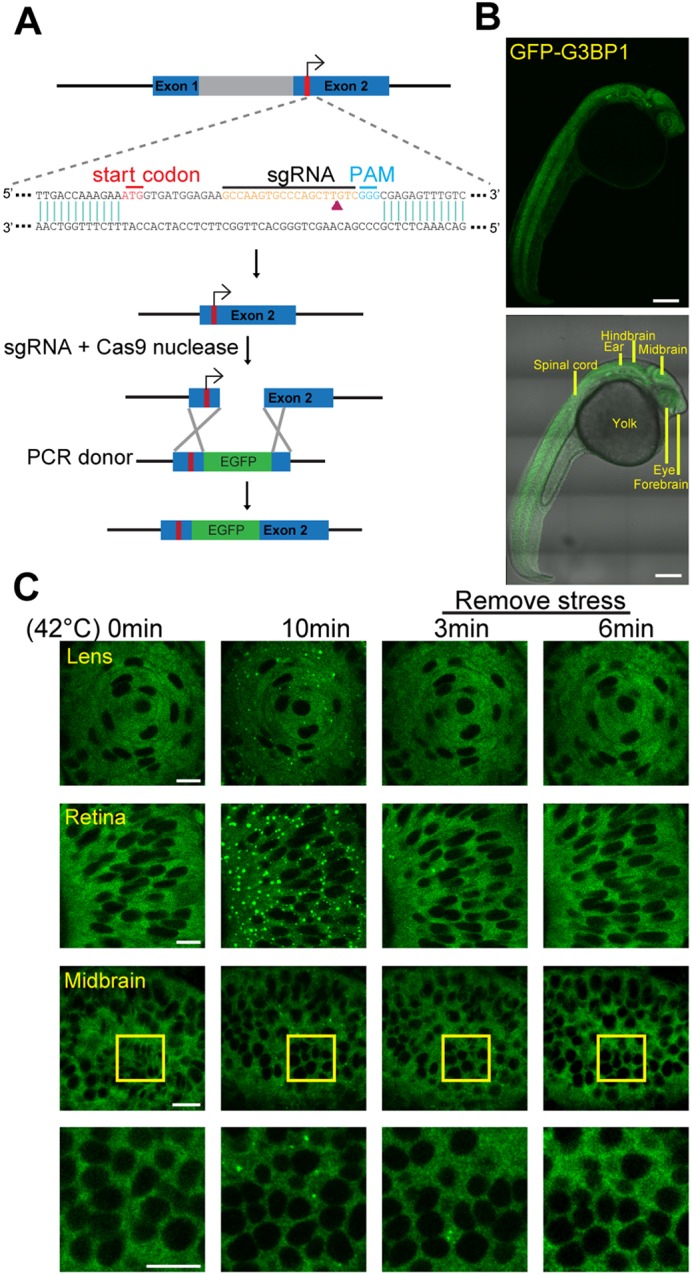
**Establishment and characterization of GFP–G3BP1 knock-in zebrafish.** (A) Schematic representation of the gene editing strategy used to insert GFP into the zebrafish *g3bp1* locus. (B) Top, *z*-stacked picture showing the expression pattern of GFP–G3BP1 in 1 dpf embryo under basal conditions. Bottom, GFP reporter signal overlapped with bright-field image. Scale bars: 200 µm. (C) Stress granules in lens, retina and midbrain cells of 1 dpf GFP–G3BP1 knock-in zebrafish when exposed to 42°C for 0 or 10 min, and at 3 and 6 min after the removal of heat stress shock. Enlarged images of the areas in the midbrain region marked by yellow squares are shown in the lower panels. Scale bars: 10 µm.

To perform gene editing in zebrafish, we microinjected sgRNA and Cas9 nuclease into zebrafish embryos to excise the zebrafish *g3bp1* gene within a 250-bp region covering either the start codon or the stop codon and then attempted to fuse the GFP sequences next to the N- or C-terminus of zebrafish G3BP1 via recombination of the donor DNA. The donor DNA was generated using PCR and contained a GFP fragment flanking two 35-bp homologous arms. This strategy has been shown to promote recombination efficiency (Paix et al., 2017). However, we were unable to fuse the GFP immediately adjacent to either the start or stop codons after numerous attempts. Therefore, we modified the strategy and directly introduced the donor DNA at the excision site nearest to the N-terminus (Fig. 1A), and were able to obtain zebrafish expressing endogenous G3BP1 with GFP inserted after the tenth residue at the N-terminus (Fig. 1B; Fig. S1A). The first 10 residues in G3BP1 are highly conserved but not required for its function as a stress granule regulator (Vognsen et al., 2011, 2013). To validate that the insertion of GFP between the tenth and eleventh residues of G3BP1 will not impair its function, we made two GFP–G3BP1 fusion constructs, one with GFP immediately after the ATG start codon (0AA-GFP–G3BP1) and the other with GFP inserted between the tenth and eleventh residues (10AA-GFP–G3BP1). We compared the stress granule formation (Fig. S1B,C,E,F) and dynamics (Fig. S1D,G) in cells transiently transfected with either of those two stress granule reporters and used heat shock (Fig. S1B,C,D) or sodium arsenite (Fig. S1E,F,G) to induce stress granule formation. There was no significant difference in the response patterns between the two constructs. Furthermore, we performed whole-genome sequencing of the F1 10AA-GFP–G3BP1 knock-in fish to examine whether GFP was erroneously inserted in other genes, and we found that *g3bp1* was the only gene tagged, indicating that the GFP reporter was unique to G3BP1 (see Materials and Methods)**.** Therefore, we proceeded to use the 10AA-GFP–G3BP1 (denoted as GFP–G3BP1 thereafter) knock-in zebrafish in our subsequent studies.

First, we characterized the expression patterns of GFP–G3BP1. Under normal basal conditions, GFP–G3BP1 was diffusely expressed in cytosol. Ten minutes of heat shock significantly increased granule formation in the lens, retina and brain. After removing stress, the granules quickly disappeared (Fig. 1C). The distribution and aggregation patterns of the zebrafish GFP–G3BP1 reporter were identical to those of previously reported cellular stress granule reporters (Tourrière et al., 2003; Wang et al., 2019) and indicated that we had successfully established an *in vivo* stress granule reporter.

**Fig. 2.**
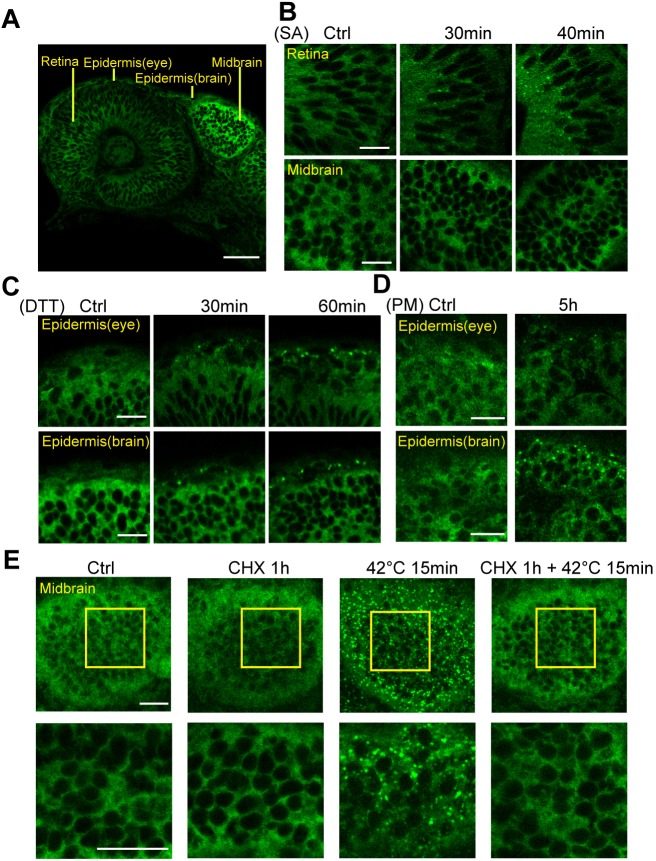
**The GFP–G3BP1 reporter responds to oxidative and ER stresses in embryonic zebrafish.** (A) Single-layer image showing GFP–G3BP1 expression in 1 dpf zebrafish under basal conditions. This region is examined in more detail in panels B and C. (B) Induction of stress granules in the retina, but not in the brain, of 1 dpf GFP–G3BP1 knock-in zebrafish after 30 mM sodium arsenite (SA) exposure in the medium for the indicated amount of time. (C) Induction of stress granules in epidermal cells in 1 dpf zebrafish exposed to 20 mM dithiothreitol (DTT) stress for indicated amount of time. (D) Induction of stress granules in the epidermal cells in 1 dpf zebrafish exposed to 10 mg/ml puromycin (PM) stress for 5 h. (E) Stress granule formation in midbrain was suppressed by treatment with 10 mg/ml cycloheximide (CHX). Enlarged images of the yellow square areas in the midbrain region are shown in the lower panels. Images show representative results from 2–3 independent experiments each with *n*=3–4 zebrafish examined at each condition. Scale bars: 20 µm.

Next, we evaluated whether the GFP–G3BP1 reporter could respond to other stress signals. Sodium arsenite (SA) is a potent stress granule-inducing agent (Matsuki et al., 2013; Parker et al., 1996; Tourrière et al., 2003) and dithiothreitol (DTT) is commonly used to induce ER stress (Lodish and Kong, 1993; Shen et al., 2002). We treated zebrafish at 1 day post-fertilization (1 dpf) with SA or DTT for 30–60 min and assessed stress granule formation using immunofluorescence microscopy (Fig. 2B,C). Fish embryos were able to endure 30 mM SA treatment for up to 40 min. Longer treatment (50 min) significantly damaged the integrity of the epidermis and caused cardiac arrest (data not shown). SA treatment was able to induce stress granules in the retina and moderately in epidermis (data not shown) but not in the brain (Fig. 2A,B). Exposure to 20 mM of DTT for up to 60 min did not affect epidermis integrity and induced the formation of stress granules in the epidermis in both the eye and midbrain regions (Fig. 2C). Therefore, the *in vivo* GFP–G3BP1 reporter was shown to respond to known stress granule-inducing agents.

Dissociation of ribosomal mRNA with puromycin could stimulate stress granule formation, while blocking the elongation of ribosomes using cycloheximide could suppress stress granule formation (Kedersha et al., 2000). To further characterize GFP–G3BP1 as an *in vivo* stress granule marker, we treated the GFP–G3BP1 knock-in zebrafish with puromycin and found increased numbers of GFP–G3BP1-positive punctate in epidermal cells in the eyes and midbrain (Fig. 2D). In contrast, cycloheximide treatment blocked the formation of stress granules in the midbrain cells of fish exposed to heat stress (Fig. 2E). Taken together, these results validated endogenous GFP–G3BP1 expression as a reliable *in vivo* marker of stress granule.

### Stress granule formation differs by brain regions

**Fig. 3.**
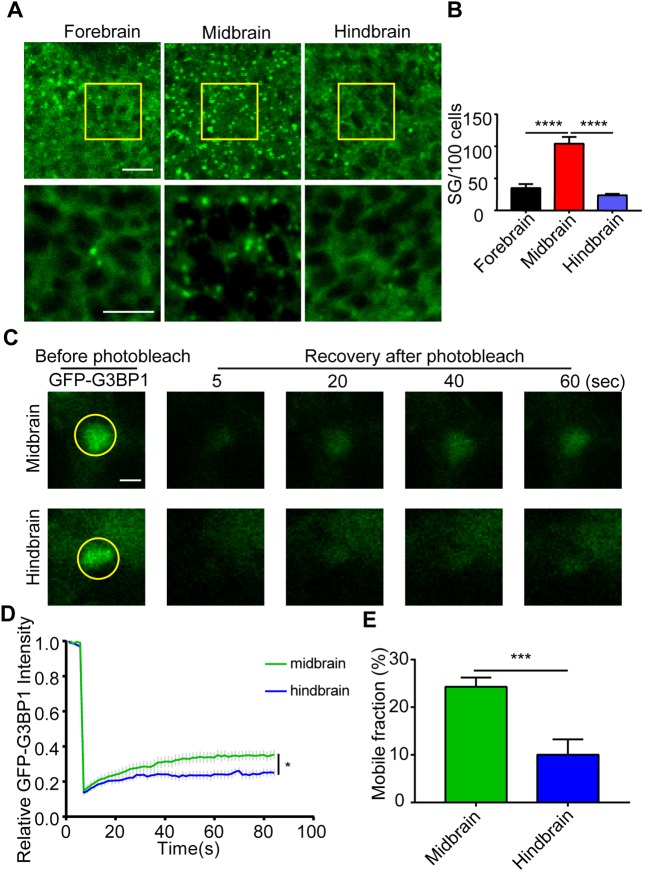
**Stress granule formation varies in different brain regions.** (A) Ten-minute heat shock-induced stress granules in the forebrain, midbrain and hindbrain region in 1 dpf GFP–G3BP1 knock-in zebrafish. The yellow boxed areas were enlarged and shown in the lower panels. Scale bars: 10 µm. (B) Quantification of stress granules (sized ≥0.1 µm) formed in cells from each region. Cells quantified were at the same depth (20 µm under the epidermis) in each region to minimize any potential difference due to heat conductance. Values represent mean±s.e.m., *n*=5 zebrafish, 100–120 cells per field. ****P≤0.0001 by unpaired Student's *t*-test. (C–E) Stress granule dynamics in heat-shocked cells from midbrain and hindbrain in 1 dpf GFP–G3BP1 knock-in zebrafish. After removal of heat shock, selected stress granules were analyzed using FRAP. All the stress granule-positive cells analyzed using FRAP were at the same depth (10 µm under epidermis). (C) Representative images of the stress granules before and after photobleaching at different times. Scale bar: 2 µm. (D) Signal intensity of GFP fluorescence from FRAP. The average fluorescence intensity before photobleaching was designated as 1. (E) Mobile fraction calculated from the FRAP analysis in D. Values in D,E represent mean±s.e.m. For each brain region, 14–15 cells from 5–6 zebrafish were analyzed. *P≤0.05, ***P≤0.001 by unpaired Student's *t*-test.

Once we had validated the reliability of the GFP–G3BP1 reporter *in vivo*, we investigated whether the stress response could vary by different brain regions during development. Zebrafish brain morphogenesis starts after the closure of the neural tube, usually at 17 hours post-fertilization (hpf) (Kimmel et al., 1995; Lowery and Sive, 2005). By 1 hpf, the indentations at the outside of the neural tube could clearly define fore-, mid- and hindbrain (Kimmel et al., 1995; Lowery and Sive, 2005). We examined the stress granules formed in brain cells in those regions under identical conditions (Fig. S2). Zebrafish at 1 dpf were exposed to 42°C heat stress for 10 min, and the brain cells at 20 µm under the epidermis were imaged and assessed for the number of stress granules (Fig. 3A). Interestingly, the number of stress granules in midbrain cells was significantly higher than in either the forebrain or hindbrain (Fig. 3B). This difference was unlikely to be caused by different GFP–G3BP1 expression levels (Fig. S2).

With the GFP–G3BP1 reporter, we were able to assess stress granule dynamics *in vivo* using fluorescence recovery after photobleaching (FRAP). Although FRAP of a stress granule component protein is a common approach for stress granule characterization, this method has never been attempted in live animals. We compared the stress granules in the midbrain and hindbrain cells at the same depth under the epidermis and found that stress granule dynamics and mobile fraction were higher in the midbrain cells (Fig. 3D,E), consistent with higher numbers of granules in those cells after heat stress. It is worth noting that compared to stress granules formed in cultured cells (Wang et al., 2019; Wheeler et al., 2016), the dynamics of stress granules in embryonic stage zebrafish brain cells were much lower. After photobleaching, fluorescence recovery reached only 30% of the original signal intensity after 80 s (Fig. 3D), indicating moderate dynamics.

### Stress sensitivity and resilience in zebrafish embryos

**Fig. 4.**
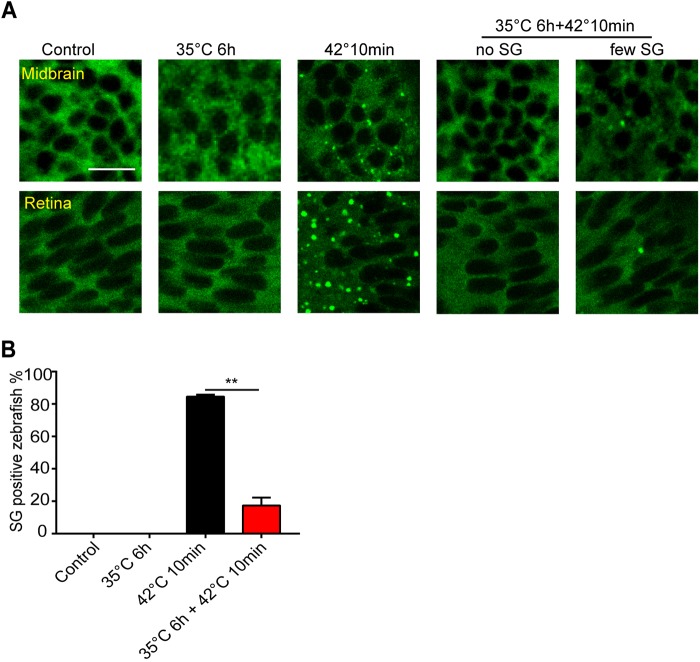
**Heat pre-conditioning suppresses stress granule formation.** (A) Representative images showing stress granule formation in midbrain and retina cells of 1 dpf GFP–G3BP1 knock-in zebrafish with indicated treatment paradigms. Control, fish kept at ambient temperature 28°C; 35°C 6 h, fish kept at 35°C for 6 h; 42°C 10 min, fish heat-shocked at 42°C for 10 min; 35°C for 6 h+42°C 10 min, fish first exposed to 35°C for 6 h, then heat-shocked for 10 min at 42°C. Scale bar: 10 µm. (B) Mean±s.e.m. percentage of zebrafish with stress granules detected in the midbrain. Data from two independent experiments, with *n*=10, 7, 19 and 17 fish for each condition. **P≤0.01 by unpaired Student's *t*-test.

It has been shown that chronic or pre-conditioning stress can limit stress granule assembly under subsequent acute stress in cultured neurons (Shelkovnikova et al., 2017). To determine the effect of pre-conditioning stress on stress granule *in vivo*, we first exposed 1 dpf zebrafish embryos to 35°C for 6 h. Unlike short exposure (10 min) at 42°C, 35°C treatment did not induce any stress granules in midbrain cells or retina (Fig. 4A). Consistent with the observation in cultured cells, 35°C pre-conditioning significantly reduced stress granule formation at 42°C (Fig. 4A,B). Therefore, chronic heat stress can diminish the fast formation of stress granules during heat shock *in vivo*.

**Fig. 5.**
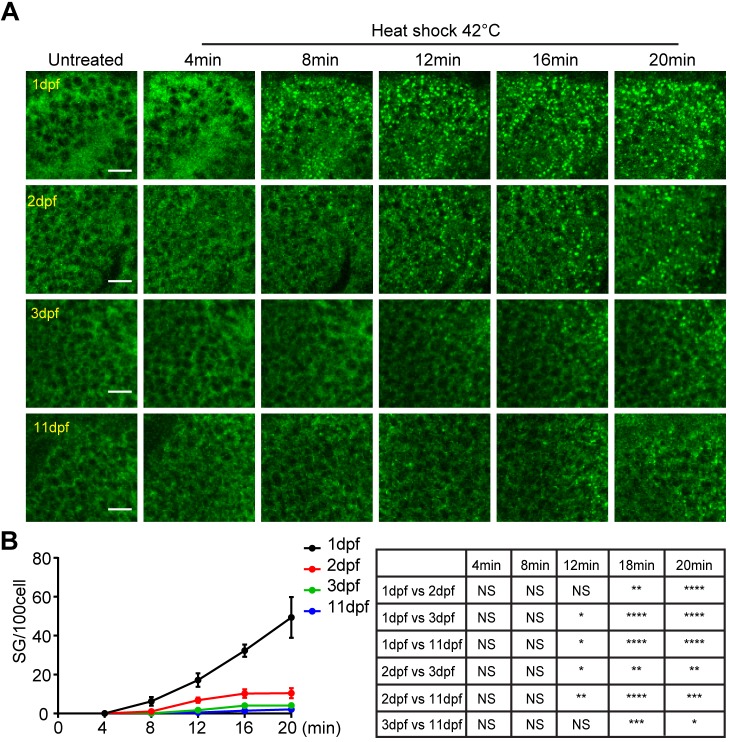
**Delayed stress granule formation in zebrafish larvae.** (A) Representative images showing the formation of stress granules in the midbrain (optical tectum) at indicated time with heat shock at 42°C for GFP–G3BP1 knock-in fish at different ages during early development. Scale bars: 10 µm. (B) Quantification of the number of stress granules (sized ≥1 µm) at the same depth (20 µm under epidermis) in fish from 1 to 11 dpf as indicated. Values represent mean±s.e.m.; *n*=4 zebrafish for each age, with 100–300 cells scored for each fish. Statistical results analyzed by two-way ANOVA followed by multiple comparison are shown in the table to the right. *P≤0.05, **P≤0.01, ***P≤0.001, ****P≤0.0001.

The *in vivo* stress granule reporter is also a great tool to determine whether stress granule formation is affected by the age of zebrafish during development. We heat-shocked (42°C) GFP–G3BP1 knock-in zebrafish of different ages (1, 2, 3 and 11 dpf) for 20 min and analyzed the stress granule formation in midbrain cells using live imaging. Surprisingly, stress granule formation was most efficient in 1 dpf embryos and gradually decreased with developmental age (Fig. 5A,B; Fig. S3). At 8 min with heat shock, granules in 1 dpf fish were clearly visible and their number consistently increased with the duration of heat shock. In contrast, for 3 dpf and 11 dpf fish larvae, only a few granules per 100 cells were detected even after 12–20 min of heat shock. Therefore, zebrafish in embryonic stages have a more efficient stress response.

**Fig. 6.**
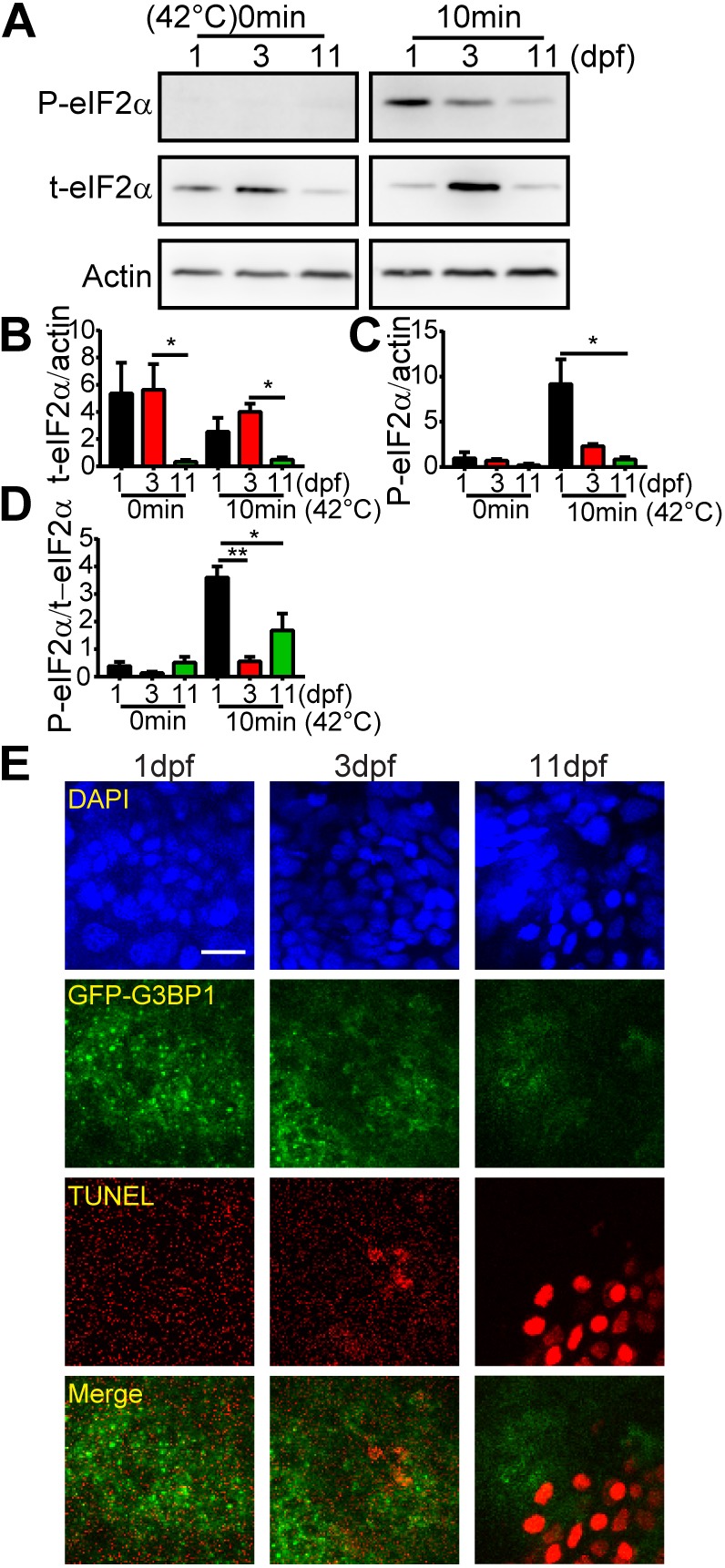
**Heat shock-induced cell death coincides with decreased level of p-eIF2α and reduced number of stress granules in fish larvae.** (A) Representative western blots showing the expression of phosphorylated eIF2α (p-eIF2α), total eIF2α (t-eIF2α) and actin after 10 min heat shock in the brain of 1, 3 and 11 dpf GFP–G3BP1 knock-in zebrafish. (B–D) Quantification of the expression of t-eIF2α relative to actin (B), p-eIF2α relative to actin (C) and p-eIF2α relative to p-eIF2α (D). Values represent mean±s.e.m. Data from three independent experiments, with *n*=15–20 zebrafish brains pooled for protein analysis for each condition. *P≤0.05, **P≤0.01 by unpaired Student's *t*-test. (E) Representative images showing stress granule formation and cell death (revealed by TUNEL staining, red) in the epidermis of 1, 3 and 11 dpf GFP–G3BP1 knock-in zebrafish exposed to 42°C for 20 min. *n*=4–5 zebrafish at each age with similar results. Scale bar: 10 µm.

To gain mechanistic insight into the differential regulation of stress granules in fish at different developmental stages, we examined the expression of phosphorylated and total eIF2α in the midbrain. The relative abundance of phosphorylated (p) and total (t) eIF2α is a determining factor in stress granule formation, with higher ratio of p-eIF2α to t-eIF2α leading to translation suppression and granule assembly (Anderson and Kedersha, 2002; Wang et al., 2019). In the absence of heat stress, the expression level of t-eIF2α in the midbrain tissues of 1 dpf and 3 dpf zebrafish embryos was almost 15 times that in 11 dpf larvae, with minimal p-eIF2α (Fig. 6A,B). With 10-min heat shock, the p-eIF2α level in 1 dpf fish embryos increased dramatically (Fig. 6A,C,D). This change most likely contributed to the abundant stress granules in 1 dpf fish during heat stress (Fig. 5). It is noted that in 3 dpf fish, the level of t-eIF2α was marginally higher than that in 1 dpf fish, while the level of p-eIF2α was much lower (Fig. 6C).

The formation of stress granules is a cellular protective mechanism during acute stress (Buchan and Parker, 2009). We have noticed that heat tolerance capacity is much lower in 11 dpf larvae than in 1 dpf embryos, demonstrated by increased incidents of cardiac arrest under heat stress (data not shown). To confirm this observation at the cellular level, we performed TUNEL labeling of epidermal cells (Fig. 6E; Fig. S4). The TUNEL signals were reverse-correlated with the abundance of stress granules in skin cells, with significant TUNEL-positive signals in 11 dpf fish. We were unable to assess TUNEL signals in midbrain cells due to poor reagent penetration (data not shown). Therefore, using the *in vivo* GFP–G3BP1 knock-in stress granule reporter fish, we have found higher heat stress resistance in zebrafish embryo than in larvae, correlated with more robust stress granule formation and significantly higher expression of p-eIF2α at the embryonic stage.

## DISCUSSION

In this study, we have established a novel *in vivo* stress granule reporter in zebrafish. This tool has the potential to elevate stress granule investigation to a new level to better understand the regulation of stress granules under various conditions. Using this new reporter, we were able to track the dynamic change of stress granules *in vivo* in different parts of the body in real-time and uncover interesting biological phenomena.

As the assembly and disassembly of stress granules is a dynamic process strongly affected by the abundance of stress granule components via liquid–liquid phase separation (Protter and Parker, 2016), reliable markers that can faithfully and efficiently trace the change of stress granules are essential. In most, if not all, studies of stress granule dynamics in cultured cells, worms or flies using live imaging, overexpression of fluorescence-tagged stress granule components is the standard practice (Kedersha and Anderson, 2007; Kedersha et al., 2008; Martin and Tazi, 2014). To achieve robust signals, cells or flies stably expressing aggregate-prone, disease-associated stress granule residents such as Fus and TDP-43 have also been used to visualize stress granules (De Graeve et al., 2019; Marrone et al., 2018). However, one clear drawback of this approach is that overexpression of these stress granule marker proteins, including G3BP1, could artificially promote stress granule formation as they are frequently nucleating proteins that facilitate the assembly of stress granules (Tsai et al., 2016). By contrast, GFP-tagged endogenous G3BP1 should faithfully reflect naturally induced levels of transcription and translation of G3BP1 and the assembly and disassembly of stress granules. With this new tool, we have made some observations that could not be achieved previously. One obvious advantage of this system is that it allowed us to observe stress granule dynamics using FRAP in live animals in various parts of the body. In this study, we have provided one example where real-time stress granule dynamics could be monitored in different brain regions after heat shock.

A surprising observation from our study is the heat stress resilience at the embryonic stage in zebrafish. In mammals, heat shock during early embryonic development usually results in deleterious consequences (Alderman et al., 2018; Edwards et al., 1997; Icoglu Aksakal and Ciltas, 2018; Menon and Nair, 2018). Heat resilience gradually increases with development and coincides with the expression of heat shock proteins (Edwards et al., 1997; Mishra et al., 2018; Walsh et al., 1997). Contrary to our initial expectation, we found that 1 dpf embryos could form stress granules much more efficiently than larvae, and are more resistant to heat stress. This phenomenon coincided with a significant increase in phosphorylated eIF2α and the absence of cell death. Previous studies have implicated corticotropin-releasing factor and heat shock proteins in the stress resistance of zebrafish during development (Alderman et al., 2018; Mishra et al., 2018). Our study has now demonstrated the involvement of eIF2α in stress granule formation as part of the stress response in early development. While post-hatching larvae and juveniles have the ability to escape from unfavorable stress, for immobile zebrafish embryos, an efficient stress response mechanism would be vital for survival.

It was also interesting to discover decreased stress granule formation in zebrafish pre-conditioned by chronic heat stress. Chronic ER stress due to abnormal proteasome and lysosomal degradation pathway activity is a feature of aging and neurodegeneration (Hetz and Saxena, 2017; Naidoo, 2009; Oakes and Papa, 2015; Shelkovnikova et al., 2017). Our *in vivo* results validated similar previous observations in cultured cells (Shelkovnikova et al., 2017), and suggested that chronic stress could weaken a cellular defense mechanism and render cells vulnerable to acute stress, such as viral infection.

Although our proof-of-concept study has demonstrated the usefulness of this *in vivo* GFP–G3BP1 reporter, the system is limited by the time window of live imaging only during embryonic, larval and juvenile stages. Long-term age-related studies will not be feasible due to the change of zebrafish anatomy with maturity. Nevertheless, the effects of various disease-related proteins, especially those encoded by genes with pathogenic mutations, on stress granule biology *in vivo* could still be assessed via genetic manipulation using this model. We recognize that even though this *in vivo* GFP–G3BP1 reporter could respond to several forms of stress, the sensitivity apparently varied greatly. While our reporter is responsive to 30 mM sodium arsenite, others have shown previously that lower concentrations cause developmental defects and can even be lethal (Fuse et al., 2016; McCollum et al., 2014). Heat stress was much more efficient to induce the formation of GFP-positive stress granules, and would be the more suitable background stress source for identifying enhancers and suppressors of stress granule formation. Although we have tested a few stress paradigms, it will be of great interest to use this system to evaluate the involvement of stress granules and their real-time regulation in animals exposed to various additional environmental and behavioral stress factors. By demonstrating the value of *in vivo* stress granule markers in zebrafish, we envision the establishment of additional *in vivo* stress granule markers using similar approach in various organisms, such as in *Caenorhabditis* *elegans*. With the development and optimization of these tools, we could have a more comprehensive understanding of the regulation and function of stress granules in adaptation, stress tolerance, survival, and its relevance to human diseases.

## MATERIALS AND METHODS

### Zebrafish

Adult zebrafish (*Danio rerio*) were maintained in the National Zebrafish Resources of China (Shanghai, China) with automatic fish housing system (ESEN, China) at 28°C following the standard protocol (Mu et al., 2012). Embryos were raised under a 14 h:10 h light:dark cycle in E2 medium (15 mM NaCl, 0.5 mM KCl, 2.7 mM CaCl_2_, 1 mM MgSO_4_, 0.7 mM NaHCO_3_, 0.15 mM KH_2_PO_4_, 0.05 mM Na_2_HPO_4_). Zebrafish handling procedures were approved by Institute of Neuroscience, Shanghai Institutes for Biological Sciences, Chinese Academy of Sciences.

### Cell culture

SH-SY5Y human neuroblastoma cells were obtained from American Type Culture Collection (ATCC, VA, USA) and cultured at 37°C in 5% CO_2_ in Dulbecco's modified Eagle's medium (Invitrogen), supplemented with 10% fetal bovine serum (Invitrogen) and antibiotics (penicillin and streptomycin, HyClone, SV30010). Cells were confirmed free of mycobacteria.

### Generation of GFP–G3BP1 knock-in zebrafish mediated by CRISPR/Cas9

CRISPR/Cas9-based gene editing techniques were used to generate GFP–G3BP1 knock-in zebrafish (Li et al., 2015; Paix et al., 2017). The sequences of sgRNAs were designed according to previously reported criteria (Chang et al., 2013), and the sequence 5′-GCCAAGTGCCCAGCTTGTC-3′ was selected as the sgRNA target in the zebrafish *g3bp1* gene. The T7 promoter–sgRNA DNA template was constructed by annealing three pairs of oligonucleotides each with sticky ends using T4 ligase. The forward and reverse sequences for the three pairs of oligos were: F1: 5′-GAATTTAATACGACTCACTATAGCCAAGTGCCCAGCTTGTCGTTT-3′, R1: 5′-GACAAGCTGGGCACTTGGCTATAGTGAGTCGTATTAAATTCC-3′; F2: 5′-TAGAGCTAGAAATAGCAAGTTAAAATAAGGCTAGTCCGT-3′, R2: 5′-GACTAGCCTTATTTTAACTTGCTATTTCTAGCTCTAAAAC-3′; F3: 5′-TATCAACTTGAAAAAGTGGCACCGAGTCGGTGCTTTTT-3′, R3: 5′-AAAAGCACCGACTCGGTGCCACTTTTTCAAGTTGATAACG-3′.

The sgRNAs were synthesized with the HiScribe T7 High Yield RNA Synthesis Kit (NEB, E2040S) and purified with the RNeasy Mini Kit (QIAGEN). The donor DNA construct contained GFP sequences flanked by two 35-bp homologous arms directed at the endogenous *g3bp1* sequences. The donor DNA was constructed via PCR using PrimeSTAR HS DNA Polymerase (Takara) and purified with the PCR Purification Kit (TIANGEN). Cas9 Nuclease (NEB, M0386S), sgRNAs and donor DNA were co-injected into the animal pole of zebrafish embryos at the one-cell stage. Each embryo was injected with 1 nl solution containing 600 ng/μl Cas9 nuclease, 30 ng/μl sgRNA and 300 ng/μl donor DNA. The embryos with fluorescence were selected and raised to adulthood. The correct transgene expression in F0 fish was validated by PCR amplification and sequencing. The forward and reverse sequences for PCR identification were: f1: 5′-GGGTGAAGAAACAGTGGAGGTGC-3′; f2: 5′-CGGCCCCGTGCTGCTGCCCGACAACC-3′; r: 5′-CACCTGTGCAGGTAGTCAGGAGCCTGG-3′.

F0 GFP–G3BP1 knock-in male fish were mated with albino (slc45a2^b4^) fish to generate F1 offspring. Whole-genome sequencing of F1 was performed by Annoroad Inc. to validate the absence of off-target insertion of GFP at locations other than at the intended site. Briefly, genomic DNA was isolated from pools of three 3 month post-fertilization F1 GFP–G3BP1 knock-in zebrafish. The GFP sequence and the zebrafish genome were designated as the reference genomes. Clean reads were mapped to the reference genomes using the Burrows–Wheeler Alignment tool (BWA). Reads that matched both the zebrafish genome and GFP were selected. Then the selected reads were re-aligned with BLAST to map the specific genetic loci in the zebrafish genome. The mapping results indicated that GFP was inserted between base pairs 25636639 and 25636640 on chromosome 14 in the *g3bp1* gene.

### Plasmid transfection

SH-SY5Y cells were transfected with GFP–G3BP1 plasmids using Lipofectamine 2000 reagent (Invitrogen) at 5 µg of DNA per 3.5-mm dish. Plasmids: 10AA-GFP–G3BP1 and 0AA-GFP–G3BP1 were generated using PCR cloning, with GFP sequences cloned after the tenth residue (10AA-GFP–G3BP1) or after the ATG start codon (0AA-GFP–G3BP1). Cells were harvested at 48 h for FRAP analysis.

### Live imaging under heat shock conditions

For live imaging of stress granules, zebrafish at different ages were individually embedded in 6 cm glass dishes in 1.5% low melting-point agarose (Sigma-Aldrich) with ventral side facing up. For 1 dpf or 2 dpf zebrafish, the embryos were first dissected from eggs before embedding. Then, heated embryo medium (15 mM NaCl, 0.5 mM KCl, 0.05 mM Na_2_HPO_4_, 0.15 mM KH_2_PO_4_, 1.0 mM CaCl_2_, 1.0 mM MgSO_4_, 0.7 mM NaHCO_3_) was pumped continuously in and out of the dish with a peristaltic pump. Medium within the dish would reach 42°C within 30 s. Time-lapse and *z*-stack images were taken under the indicated conditions with a confocal microscope (Nikon NiE) with 25× water-immersion lens. The resolution of all the images was 1024×1024 pixels.

### Drug treatment

Zebrafish embryos at 1 dpf were dissected from the eggs and then soaked in drug solution for indicated amount of time (30–60 min). Subsequently, the embryos were fixed with 4% paraformaldehyde for 24 h and then transferred to PBS for imaging. The concentrations of sodium arsenite (S7400-100G, Sigma-Aldrich), DTT (Sigma-Aldrich) and puromycin (A11138, Sigma-Aldrich) were 30 mM, 20 mM and 10 mg/ml, respectively. For the cyclohexamide (CHX) experiments, 1 dpf zebrafish were treated with 1 mg/ml CHX for 1 h, followed by 42°C heat shock with the addition of CHX for 10 min, then fixed with 4% PFA for 24 h for imaging.

### Fluorescence recovery after photobleaching

For fluorescence recovery after photobleaching (FRAP) experiments in zebrafish, 1 hpf zebrafish embryos were first dissected from eggs. The embryos were soaked in 42°C embryo medium and embedded in 6 cm glass dishes in 1.5% low melting-point agarose (Sigma-Aldrich) with the ventral side up. Stress granules were photobleached and GFP intensity was measured before and after bleaching.

For FRAP experiment in SH-SY5Y cells, cells were transfected with GFP–G3BP1 reporter plasmids. At 48 h after transfection, cells were treated with 20 µM sodium arsenite for 30 min to induce stress granules. Stress granules were photobleached and GFP intensity was measured before and after bleaching as described (Wang et al., 2019).

### Pre-conditioning heat stress

For pre-conditioning heat stress, 1 dpf zebrafish embryos were raised at a basal temperature of 28°C, removed from eggs and soaked in 35°C embryo medium for 6 h, and then transferred to 42°C embryo medium for 10 min. Subsequently, the heat-shocked embryos were fixed in 4% paraformaldehyde for 24 h and then transferred to PBS for imaging.

### Confocal imaging

Time-lapse and *z*-stack images were taken under the indicated conditions. Images were taken with a Nikon NiE-A1 confocal microscope with 25× water-immersion lens or Nikon FN1 confocal microscope with 60× water-immersion lens (for FRAP). The resolution of all the images was 1024×1024 pixels.

### Western blotting

Zebrafish embryos and larvae were incubated at 42°C for 10 min, and the midbrain tissues were dissected and lysed in RIPA buffer (150 mM NaCl, 50 mM Tris pH 8.0, 1% NP40, 1% sodium deoxycholate, 0.1% SDS) supplemented with protease inhibitor cocktail (Roche) and phosphatase inhibitor cocktail (Roche). Proteins were resolved using SDS-PAGE, and the protein bands were visualized using Bio-Rad western ECL substrate kit. The band intensity in immunoblots was determined using Bio-Rad Quantity One software. The primary antibodies used were: mouse anti-eIF2α (sc-133132 Santa Cruz Biotechnology, 1:1000); rabbit anti-phospho-eIF2α (9721 Cell Signaling Technology, 1:1000); mouse anti-actin (M20010 Abmart, 1:5000). Secondary antibodies used were: goat anti-rabbit IgG-HRP (Abmart M21002L, 1:5000) and goat anti-mouse IgG-HRP (Abmart M21001L, 1:5000).

### TUNEL assay

Zebrafish at different ages were heat-shocked for 20 min, then fixed in fresh 4% paraformaldehyde in PBS overnight at 4°C and dehydrated using methanol (3×10 min). Zebrafish were further permeabilized in a solution containing 0.1% Triton X-100 and 0.1% sodium citrate in PBS for 1 h at room temperature followed by rinses in PBS (2×10 min). The samples were subjected to TUNEL assay using the TMR-RED *in situ* cell death detection kit (Roche, Basel, Switzerland) according to the manufacturer's protocols, and then rinsed in PBST (PBS, 0.3% Tween) (3×15 min). Samples were also stained with DAPI for 10 min with rinses in PBST (2×10 min) to label nuclei. Images were taken with a confocal microscope (Nikon NiE) with 25× water-immersion lens.

### Quantification and statistical analysis

The number of zebrafish used in each experiment is described in figure legends. For data analysis, the results are presented as the mean±s.e.m., with statistical significance analyzed using Student's *t*-test or two-way ANOVA, using GraphPad Prism 5. The statistical test for each experiment is indicated in figure legends (*P≤0.05; **P≤0.01; ***P≤0.001; ****P≤0.0001).

## Supplementary Material

Supplementary information
