# Supplementary material for: Heat resilience in embryonic zebrafish revealed using an in vivo stress granule reporter
Source: J Cell Sci. 2019 Oct 18;132(20):jcs234807. doi: 10.1242/jcs.234807 (PMC6826007; doi:10.1242/jcs.234807)
Supplement: Supplementary information [file joces-132-234807-s1.pdf]

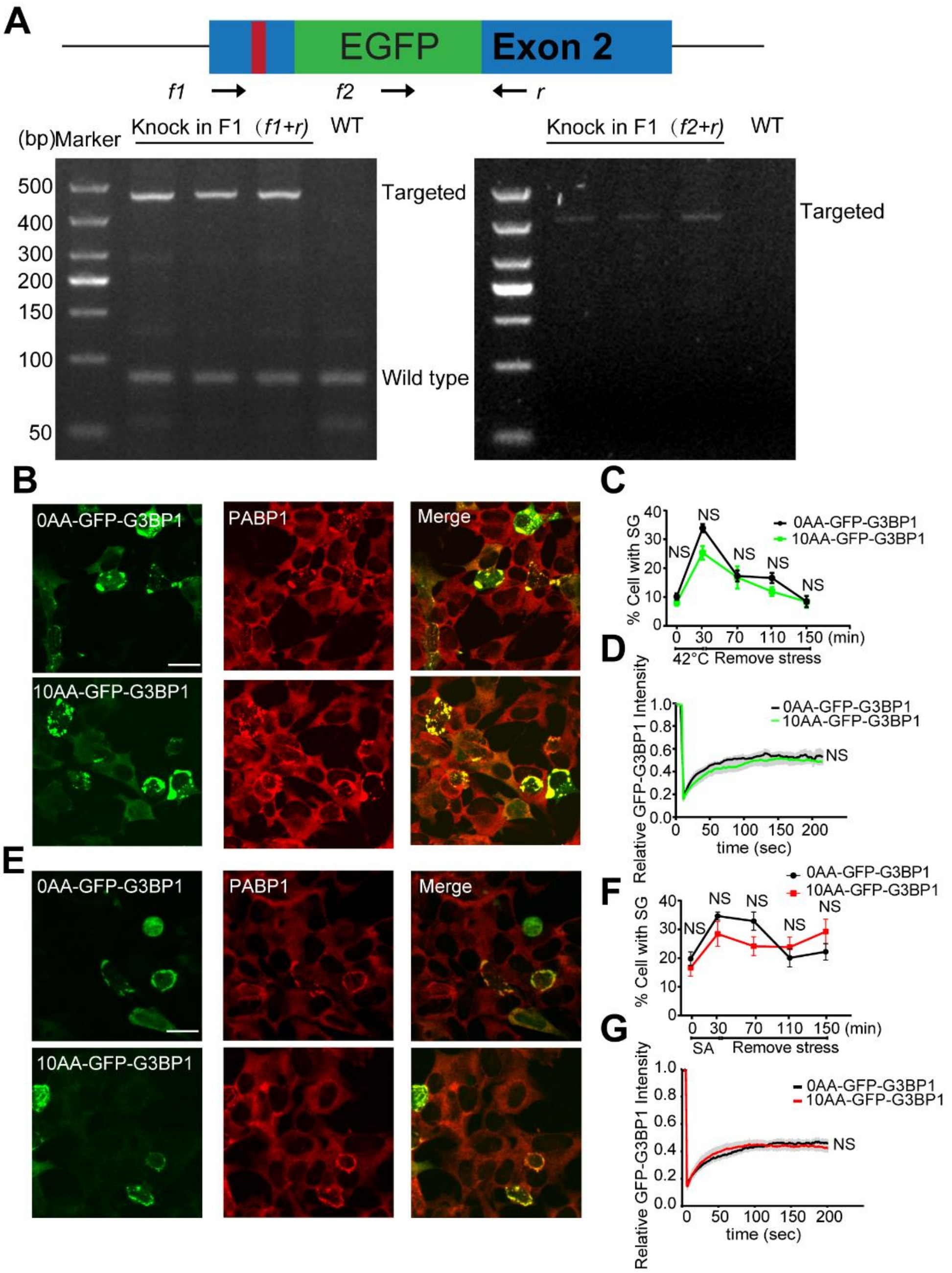

Fig S1. Validation of GFP-G3BP1 KI zebrafish

(A)Genotyping strategy and results from two sets of primers (*f1+r and f2+r*).

(B) Representative images showing the same response of 0AA-GFP-G3BP1 or 10AA-GFP-G3BP1 to heat shock stress (42°C for 30 minutes) in SH-SY5Y cells. (C) Stress granule formation in SH-SY5Y cells transfected with either 0AA-GFP-G3BP1 or

Journal of Cell Science • Supplementary information

10AA-GFP-G3BP1 reporter plasmid after heat shock at 42°C for 30 minutes. Cells were fixed and immunolabeled at indicated time after removal of stress for stress granule imaging and quantification (mean ± S.E.M.; n=10 fields for each time point, at least 10-15 GFP positive cells per field. (D) FRAP analysis of heat shocked SH-SY5Y cells expressing 0AA-GFP-G3BP1 or 10AA-GFP-G3BP1. Cells were heat shocked at 42°C for 30 minutes, and the stress granules dynamics were analyzed by FRAP after stress removal (mean ± S.E.M.; n = 7-8 cells per sample, by unpaired Student's-*t*-test). (E) Representative images showing the same response of 0AA-GFP-G3BP1 or 10AA-GFP-G3BP1 to sodium arsenite stress (20μM for 30 minutes) in SH-SY5Y cells. (F) SH-SY5Y cells transfected with either reporter plasmid were stress shocked with 20μM sodium arsenite for 30 minutes and fixed at indicated time after removal of stress for stress granule imaging and quantification (mean ± S.E.M.; n=10 fields for each time point, at least 10-15 GFP positive cells per field. (G) FRAP analysis of SH-SY5Y cells expressing 0AA-GFP-G3BP1 or 10AA-GFP-G3BP1. Cells were treated SA as described above. After stress was removed, stress granules dynamics were analyzed by FRAP. For FRAP the average fluorescence before photobleaching was designated as 1 (mean ± S.E.M.; n = 8-9 cells per sample, by unpaired Student's-*t*-test). (Scale bar =10μm)

Journal of Cell Science • Supplementary information

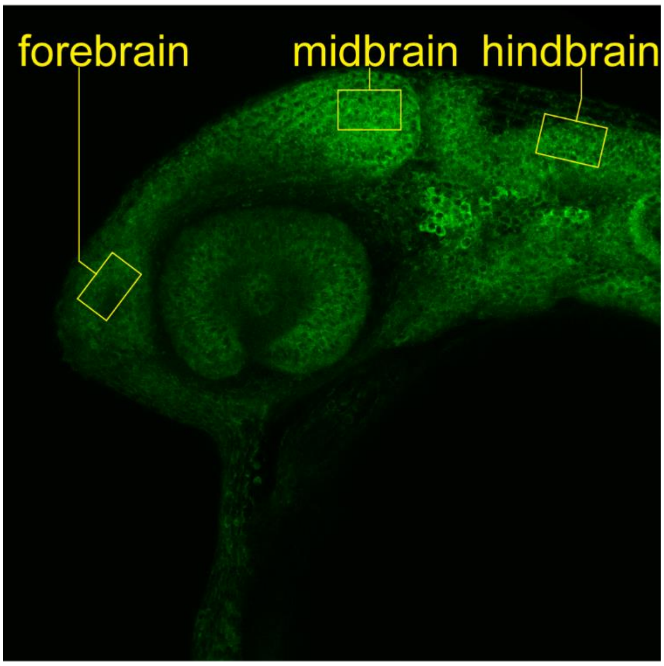

Fig S2. The forebrain, midbrain, hindbrain regions in 1dpf fish embryo selected for SG formation and dynamics analysis. 1dpf zebrafish at normal condition was fixed in 4% PFA for imaging.

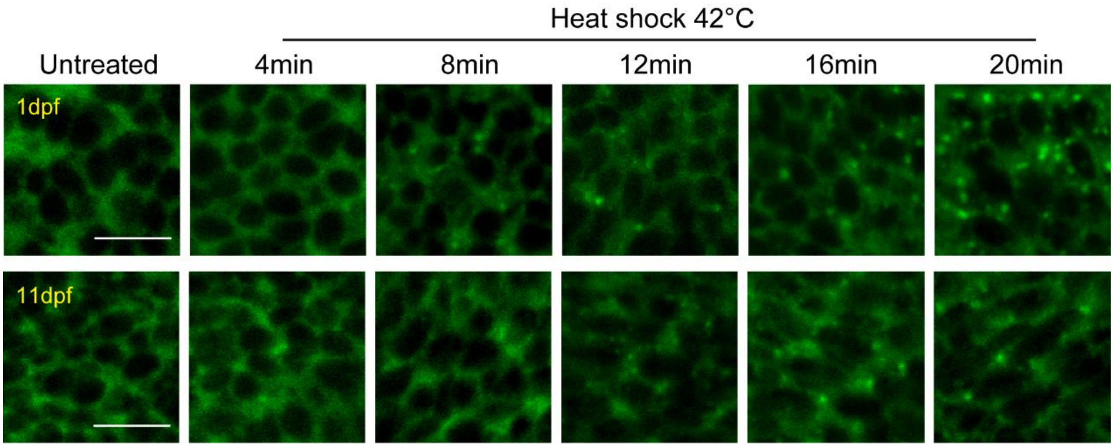

Fig S3. Delayed stress granule formation in zebrafish larvae

Enlarged images of the midbrain region of 1dpf and 11dpf zebrafish at various time points after heat shock as shown in Fig 5A. Scale bar =10μm

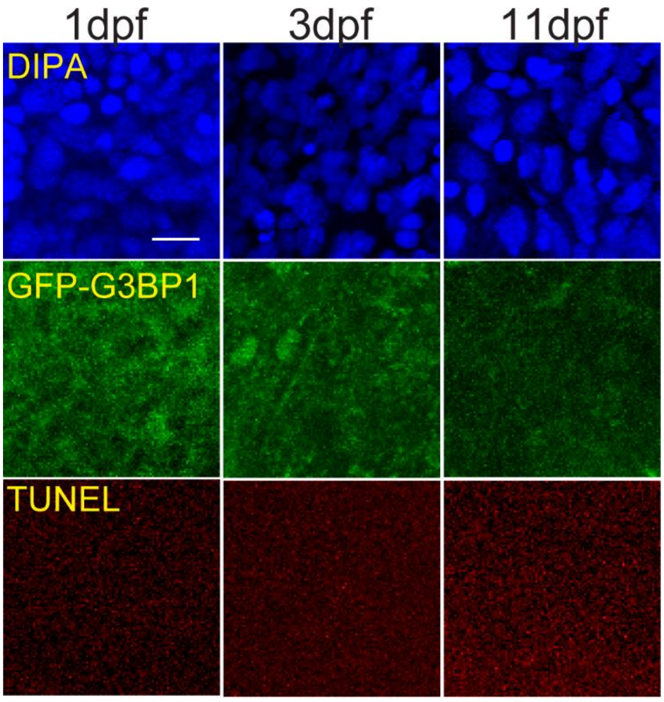

Fig S4. Absence of cell death in zebrafish kept at ambient condition. Zebrafish of different age kept at 28 °C were fixed in 4% PFA for TUNEL assay.
